# Management Considerations for an Isolated Enlarged Cavum Septum Pellucidum: A Case Report

**DOI:** 10.7759/cureus.95963

**Published:** 2025-11-02

**Authors:** Kebereab Feyissa, Iustin Scobercea, Gerald J Valentini, Stephen J Despins

**Affiliations:** 1 Physical Medicine and Rehabilitation, Liberty University College of Osteopathic Medicine, Lynchburg, USA; 2 Sports Medicine/Physical Medicine and Rehabilitation, Collaborative Health Partners Specialty Services, Lynchburg, USA; 3 Osteopathic Manipulative Medicine, Liberty University College of Osteopathic Medicine, Lynchburg, USA

**Keywords:** cavum septum pellucidum, fetal anomalies, physical medicine and rehabilitation, radiology, ultrasound anatomy

## Abstract

We present the case of a male infant identified prenatally with an isolated enlarged cavum septum pellucidum (CSP) on ultrasound with otherwise normal neuroanatomy. At 24 weeks and four days of gestation, an anatomy scan demonstrated a CSP measuring 7.5 mm (95th percentile). A repeat maternal-fetal medicine ultrasound at 26 weeks and four days revealed further enlargement to 9.3 mm. While the CSP size was above average, no other structural anomalies were identified. The parents were counseled on potential associations, including chromosomal abnormalities and neurodevelopmental disorders. The pregnancy and delivery were otherwise uncomplicated. At 17 months of age, the child has met or exceeded gross motor, fine motor, language, and social-emotional developmental milestones. This case emphasizes the importance of distinguishing isolated CSP enlargement from cases with additional anomalies, providing appropriate counseling, and ensuring postnatal developmental follow-up.

## Introduction

The cavum septum pellucidum (CSP) is a normal, midline, fluid-filled cavity located between the two leaflets of the septum pellucidum, situated between the frontal horns of the lateral ventricles [1]. It is a normal finding in fetuses and neonates, but persistence into adulthood is considered an anatomic variant [1]. It is typically visualized in the fetal brain between 18 and 37 weeks of gestation and generally obliterates between three and six months after birth [1,2]. Although its exact physiological role remains uncertain, proposed functions include serving as a cerebrospinal fluid reservoir, a developmental marker of commissural maturation, or an anatomical variant without clinical significance [3]. Further, it has been hypothesized to play a role in limbic system modulation due to its anatomic relationship to and connections with the fornix, hippocampus, and hypothalamus, potentially influencing emotional regulation and memory processing [3].

CSP abnormalities typically involve its absence (often associated with severe congenital brain malformations such as agenesis of the corpus callosum or septo-optic dysplasia), or its persistent enlargement into postnatal life [4,5]. A persistent or enlarged CSP has been reported in association with neurodevelopmental and psychiatric conditions, including schizophrenia, autism spectrum disorder, attention-deficit/hyperactivity disorder, and traumatic brain injury [4,5].

Unique to our case, the isolated enlargement (without additional fetal abnormalities) of the CSP in the prenatal period is poorly represented in current medical literature. We present the case of an isolated enlarged CSP detected on prenatal ultrasound with normal postnatal developmental outcomes through 17 months of age.

## Case presentation

**Figure 1:**
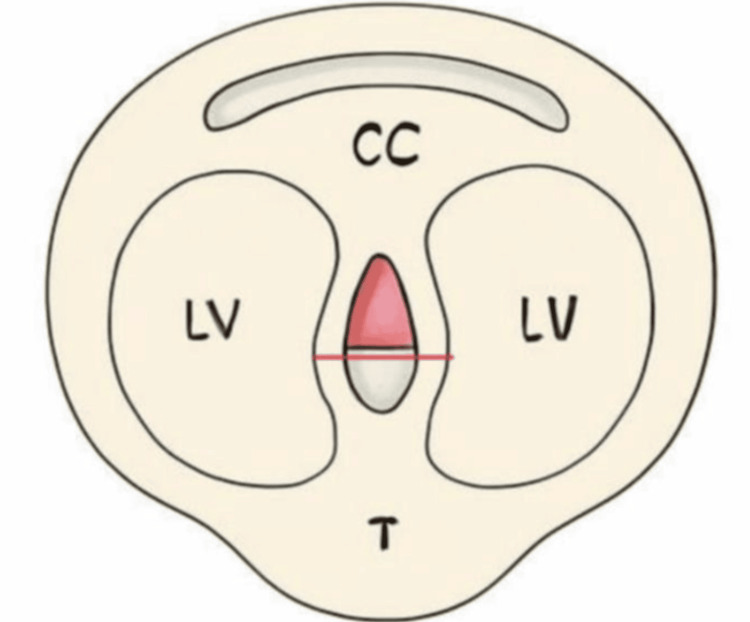
Stylized schematic of the CSP in the axial plane depicting anatomical landmarks: CC, LV, and T The CSP measurement line is indicated in red. Original image of the authors. CC: corpus callosum; LV: lateral ventricle; T: thalamus; CSP: cavum septum pellucidum

**Figure 2:**
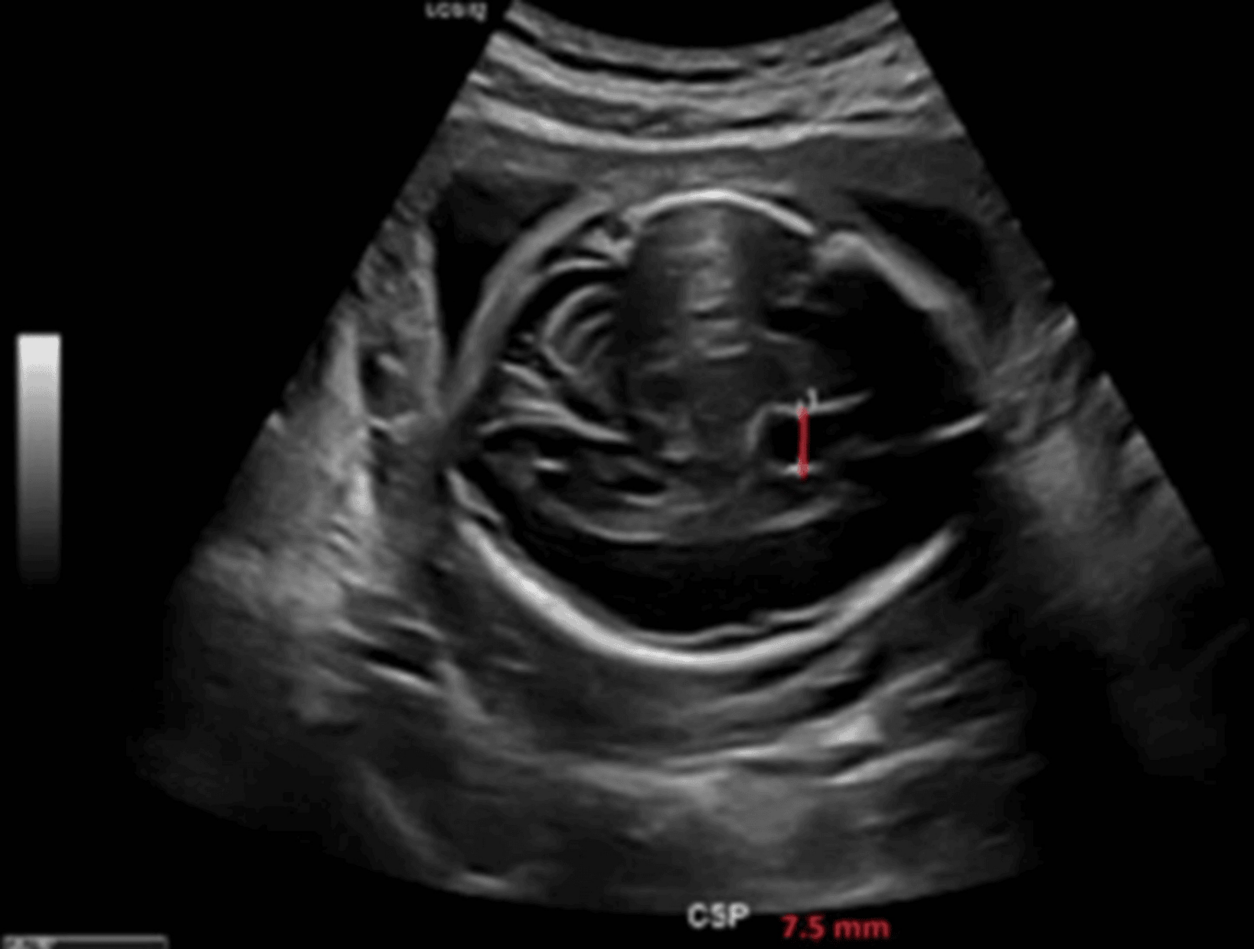
Ultrasound view of the fetal brain at 24 weeks and 4 days of gestation demonstrating the CSP (red line) measuring 7.5 mm, corresponding to the 95th percentile for gestational age CSP: cavum septum pellucidum

A male fetus, the son of one of the authors, was identified on routine anatomy ultrasound at 24 weeks and four days of gestation with an enlarged CSP measuring 7.5 mm (Figures 1, 2), corresponding to the 95th percentile for gestational age. The pregnancy was otherwise uncomplicated, and no other structural abnormalities were noted on the scan.

**Figure 3:**
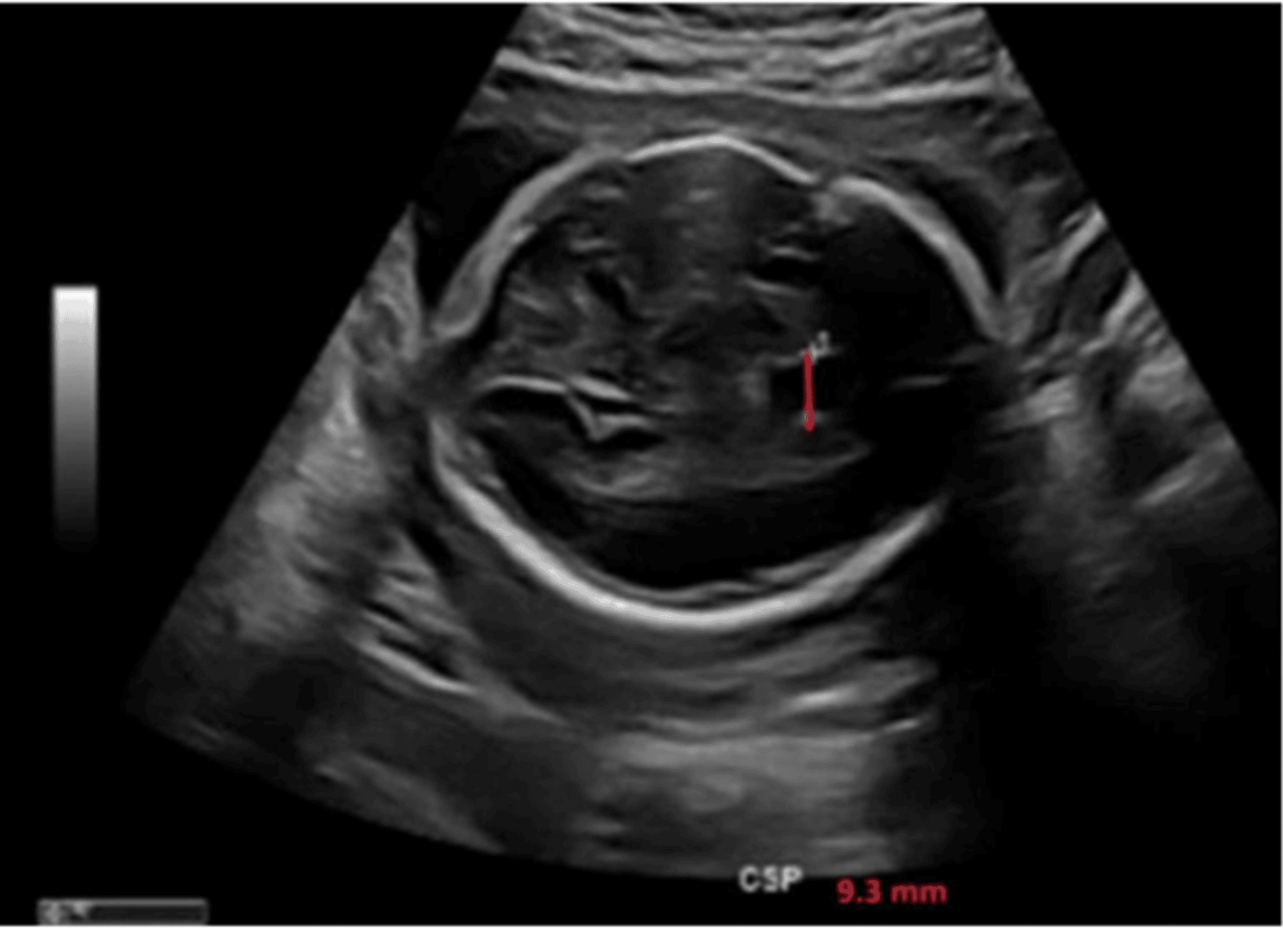
Repeat targeted ultrasound at 26 weeks and 4 days of gestation demonstrating interval enlargement of the CSP to 9.3 mm (red line). Other midline structures remain normal CSP: cavum septum pellucidum

Two weeks later, a maternal-fetal medicine (MFM) consultation and repeat targeted ultrasound demonstrated further CSP enlargement to 9.3 mm (Figure 3). The remainder of the fetal neuroanatomy (corpus callosum, ventricles, posterior fossa structures, and gyral pattern) was normal. The parents were counseled that while this finding may represent a normal variant, the medical literature suggests an increased risk for aneuploidy and 22q11.2 deletion syndrome [6]. Given the absence of additional anomalies and the otherwise reassuring ultrasound, invasive genetic testing (including amniocentesis with chromosomal microarray analysis to evaluate for submicroscopic chromosomal abnormalities, including 22q11.2 deletion syndrome) was deferred by the parents.

**Figure 4:**
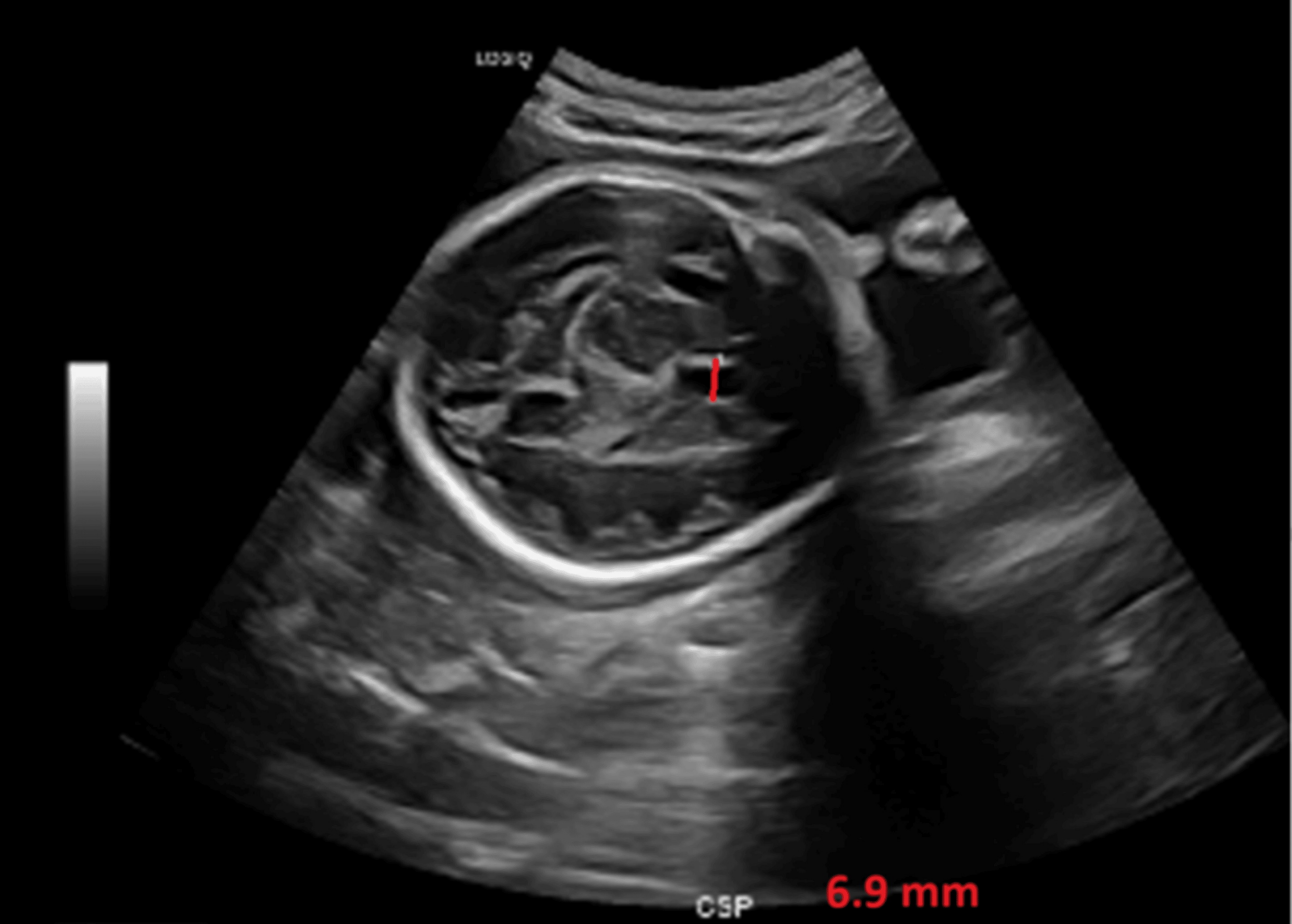
Follow-up ultrasound at 29 weeks demonstrating the CSP (red line) measuring 6.9 mm, with resolution of the previously noted enlargement CSP: cavum septum pellucidum

Three weeks later, a follow-up MFM consultation was performed, and the CSP was reassessed at 6.9 mm (Figure 4), showing resolution of the prior enlargement. However, the parents were counseled that the previously discussed increased risk for aneuploidy and 22q11.2 deletion syndrome [6] remained, as this risk is associated with any history of CSP enlargement, even if it resolves.

After an uncomplicated pregnancy, the infant was delivered at term without complications, with normal birth weight and appearance, pulse, grimace, activity, and respiration scores. Postnatal physical examination was unremarkable, and no neurological deficits were observed, including normal tone, reflexes, coordination, and responsiveness. At 17 months of age, the child had undergone developmental surveillance at all recommended pediatric well-child visits, including two weeks, one month, two months, four months, six months, nine months, 12 months, and 15 months, which indicated the child had met or exceeded expected milestones across gross motor, fine motor, language, and social-emotional domains. Assessment included structured observation and parent-report measures, with evaluation of gross motor skills such as independent walking, climbing, and throwing; fine motor skills including pincer grasp and block stacking; language abilities through vocabulary and comprehension tasks; and social-emotional behaviors via play-based interactions. Standardized developmental screening tools were utilized, including the Ages & Stages Questionnaires (Third Edition) [7] for communication, motor, and personal-social domains; the Ages & Stages Questionnaires: Social-Emotional [8] for social-emotional development; and the Denver Developmental Screening Test II [9] for a comprehensive, clinician-administered assessment.

## Discussion

The CSP is a normal midline structure of the fetal brain, typically present during mid-gestation and subsequently closing by late fetal or early postnatal life [10]. Enlargement of the CSP during the prenatal period has been a subject of clinical concern, primarily due to its potential associations with neurodevelopmental abnormalities, chromosomal anomalies, and structural central nervous system (CNS) malformations [10,11]. Despite these associations, the mechanism of CSP enlargement remains incompletely understood [10,12]. Several hypotheses have been proposed, including transient developmental delay in septal leaflet fusion, altered cerebrospinal fluid dynamics within the ventricular system, or benign anatomic variation without pathological significance [10,12].

Thresholds for defining an enlarged CSP vary among studies, but measurements exceeding the 95th percentile for gestational age are frequently utilized as a diagnostic standard [10]. Detection of CSP enlargement during prenatal imaging necessitates a careful distinction between isolated and non-isolated findings [10]. In cases where CSP enlargement coincides with additional CNS or extracranial anomalies, broader evaluation (including advanced imaging and genetic counseling) is often indicated. When identified in isolation, however, the clinical significance of CSP enlargement is less clear, and the paucity of published data limits the development of evidence-based management strategies.

Our case adds to the limited body of literature documenting isolated CSP enlargement without coexisting abnormalities. A comprehensive literature search yielded only one retrospective study [13] specifically addressing this entity. In that study of 48 fetuses with isolated dilated CSP, no chromosomal abnormalities were detected [13]. Nevertheless, 12.5% of the children were later diagnosed with neurodevelopmental delays, underscoring the possibility of adverse outcomes even in the absence of syndromic or structural anomalies [13]. Interestingly, the observed delays were not correlated with gestational age at diagnosis, CSP width, persistence of the dilatation, or other categorical variables [13]. These findings suggest that isolated CSP enlargement represents a heterogeneous entity, in which some cases may follow a benign course while others may predispose to developmental challenges [13].

In our case, enlargement of the CSP was noted at 24 and 26 weeks of gestation, and the parents were counseled about possible associations with structural brain abnormalities and chromosomal anomalies. They opted against invasive prenatal genetic testing. Following normal delivery, only pediatric developmental milestones were monitored until the age of 17 months. No further radiological assessments (including magnetic resonance imaging), corpus callosum measurements, or genetic testing were performed. The absence of postnatal imaging represents a limitation, as CSP dynamics beyond birth were not evaluated.

The uniqueness of our case lies in its characterization of isolated CSP enlargement in a setting where no additional abnormalities were demonstrated across sequential imaging and postnatal follow-up. Given the rarity of such reports, our findings contribute supplemental evidence that isolated CSP enlargement may not uniformly lead to an adverse outcome. This case highlights broader considerations regarding prenatal counseling and perinatal management. Identification of CSP enlargement, even in isolation, places parents and clinicians in a position of uncertainty, where the rarity of published outcomes complicates prognostic discussions. The current evidence suggests that, while routine karyotyping may not be warranted for isolated cases, longitudinal developmental monitoring after birth is advisable. Collaboration among MFM specialists, pediatric neurologists, and developmental pediatricians may provide the most comprehensive framework for patient care. In conclusion, our case reinforces that isolated CSP enlargement may follow a favorable course, but the brevity of follow-up, absence of postnatal radiological or genetic confirmation, and reliance on a single case warrant cautious interpretation. A probable benign outcome can be anticipated, but long-term surveillance remains essential, and further multi-institutional data are necessary to refine management and counseling strategies.

## Conclusions

This case underscores that an isolated enlarged CSP on prenatal imaging without associated brain or systemic abnormalities may represent a benign variant, but caution is warranted given the limited data, short duration of follow-up, and absence of postnatal radiological or genetic confirmation in this and similar cases. In such cases, reassurance can be provided while emphasizing the value of developmental follow-up. Evidence-based counseling helps avoid unnecessary parental anxiety while ensuring that children receive timely evaluation if concerns arise. Further research into the natural history and long-term neurodevelopmental outcomes of isolated enlarged CSP is warranted to further refine management guidelines.
